# Serum Fructosamine, Total Cholesterol, and High-Density Lipoprotein in Children with Asthma during Glucocorticoid Treatment

**DOI:** 10.5402/2011/295124

**Published:** 2011-08-14

**Authors:** A. J. Schou, O. D. Wolthers

**Affiliations:** Children's Clinic Randers, Dytmaersken 9, 8900 Randers C, Denmark

## Abstract

*Background/Aims*. Glucocorticoids may have adverse effects on carbohydrate and lipid metabolism. The present study was conducted to investigate possible effects on carbohydrate and lipid metabolism of inhaled and oral glucocorticoids in children with asthma. *Methods*. Two randomised controlled trials with blinded crossover designs were performed. Active treatment was 400 **μ**g inhaled budesonide or 5 mg prednisolone orally daily during one week. The budesonide trial included 17 and the prednisolone trial 20 school children. Serum fructosamine, total cholesterol and high-density lipoprotein were assessed. *Results*. Serum fructosamine was increased during active treatment (prednisolone 252.3 **μ**M versus placebo 247.3 **μ**M; *P* = 0.03 and budesonide 228.1 **μ**M versus no treatment 223.1 **μ**M; *P* = 0.02). Total cholesterol and high-density lipoprotein were not statistically significantly increased. *Conclusion*. Short-term treatment with oral prednisolone and inhaled budesonide may adversely affect mean blood glucose concentration. Possible long-term consequences require further investigations.

## 1. Introduction

Asthma is the most common chronic childhood disease [1]. Inhaled glucocorticoids are widely recommended as first-line treatment [2], and short-term treatment with oral glucocorticoids is often used during exacerbations [3]. Exogenous glucocorticoids—inhaled as well as oral—may cause a wide range of dose-dependent adverse systemic effects [4, 5]. These include suppression of hypothalamic-pituitary-adrenal function, growth retardation, catabolic effects on bone metabolism, suppressive effects on connective tissue including atrophy of the skin, cataracts, haematological effects, and, finally, but not least, metabolic effects [5, 6]. The metabolic effects include elevated serum glucose concentration and hyperlipidaemia, both of which are closely linked to the “Metabolic Syndrome” with a greatly increased risk for cardiovascular disease, stroke, and type II diabetes [7, 8]. Such glucocorticoid-induced effects are mediated by binding to receptors in the liver and in muscle as well as adipose tissue and may partly be due to reduced insulin sensitivity [7–9]. Little attention has been paid to the possible effects of glucocorticoid treatment on carbohydrate and lipid metabolism in children despite potentially very harmful long-term consequences. The effects of inhaled glucocorticoids which often may be used during many years in the treatment of asthma have been studied few times in adults [10–12] and once in children [13]. These studies were flawed by methodological shortcomings, and the results were largely conflicting [10–13]. The study conducted in children suggested effects of high-dose treatment with inhaled glucocorticoids on carbohydrate as well as on lipid metabolism [13]. There are no available studies of the effects of oral glucocorticoid treatment on neither carbohydrate nor lipid metabolism in children. We conducted the present study to assess effects of short-term treatment of inhaled and oral glucocorticoid treatment on carbohydrate and lipid metabolism in children as measured by serum fructosamine, an indicator of the average serum glucose concentration during the preceding 2-3 weeks [14], serum total cholesterol, and high-density lipoprotein (HDL).

## 2. Patients and Methods

Two parallel clinical trials with randomised, blinded, crossover designs were conducted. Both trials included two treatments, run-in and washout periods of one-week duration. In one trial 5 mg prednisolone once daily in the evening was compared with placebo, in the other 200 *μ*g budesonide from a dry powder inhaler (Spirocort Turbuhaler) twice daily was compared with no treatment. The sequence of active versus placebo and no treatment, respectively, was allocated by a computerized randomization scheme prepared in balanced blocks. Only study medication and inhaled *β*_2_-agonist (Bricanyl Turbuhaler 0.5 mg) as rescue medication were allowed throughout the study. 

The two studies were designed to investigate a wide range of systemic effects during glucocorticoid treatment, including short-term growth of the lower leg assessed by knemometry [15]. The power calculations were based on previous knemometry data and showed that to be able to detect a 50% reduction in lower leg growth rate with 80% probability, 16 children had to complete the study [16, 17]. Thus, the prednisolone and budesonide trials included 20 and 17 children with asthma, respectively. All children had suffered from asthma for more than one year and had been treated with inhaled glucocorticoids at least 6 months prior to inclusion. Demographics are given in Table 1. 

Blood samples were collected at the last day of the treatment periods at the same time of the afternoon ±30 minutes, centrifuged 10 min at 4000 rpm, and stored at −80°C. The children were nonfasting at the time of sampling. At the completion of both trials, serum fructosamine was batch assayed by means of a commercially available colorimetric assay with a reproducibility of 0.9% (Roche). Total cholesterol and HDL were assessed using commercially available enzyme assays (CHOD-PAP for total cholesterol with a coefficient of variation (CV) of 1.9% and direct assay for HDL with a CV of 2.7%) from Roche on a Hitachi 917. 

Pulmonary function tests were performed with the Vitalograph at each visit with measurement of forced vital capacity (FVC), forced expiratory volume in 1 second (FEV_1_), and peak expiratory flow (PEF). Throughout the trials, all children performed daily measurement of PEF in the morning and in the evening with the Mini-Wright peak-flow meter and recorded the results in asthma diaries along with symptom scores on a scale from 0 to 3 and use of *β*_2_-agonist.

The trials were conducted in accordance with international guidelines issued by the European Commission in 1990 and the Declaration of Helsinki and were approved by the local ethics committee. Informed consent was obtained from all children and their parents before inclusion.

## 3. Statistics

Serum fructosamine, total cholesterol, HDL, PEF, and FEV_1_ were compared between the treatment periods in both trials by means of a paired *t*-test. Asthma symptom scores and use of *β*_2_-agonists were analyzed by Wilcoxon rank sum test. The 5% level of significance was used. All data were tested for period and carry-over effects as described for crossover designs [18].

## 4. Results

All included children completed both trials. Serum fructosamine, total cholesterol, and HDL and the results of the statistical tests are given in Table 2. Serum fructosamine was increased during active treatment in both trials. Though numerically increased total cholesterol and HDL were not statistically significantly increased during prednisolone treatment. Small and insignificant differences were observed in total cholesterol and HDL during budesonide treatment. No period or carry over effects were detected in fructosamine, total cholesterol, or HDL. 

No significant differences were found in any parameter of pulmonary function in any of the trials.

## 5. Discussion

Carbohydrate metabolism is regulated by a wide range of complex systems [19]. Insulin is one of the primary factors in the regulation of the blood glucose concentration [19]. Insulin secretion is stimulated by glucose and other energy rich substances in the blood, that is, the higher the blood glucose, the higher the insulin secretion in healthy subjects [19]. Insulin subsequently lowers blood glucose by stimulating uptake of glucose in fat and muscle tissue and by stimulating glycogen synthesis and inhibiting glycogenolysis in the liver [19]. Endogenous cortisol counteracts insulin and increases blood glucose by inhibiting uptake into muscle and fat tissue and by increasing glycogenolysis and gluconeogenesis in the liver [7]. These effects are mimicked by prednisolone and budesonide which bind to glucocorticoid receptors and, thus, may increase mean blood glucose [20, 21]. However, in healthy subjects, a compensatory increase in insulin secretion will tend to reduce mean blood glucose to normal or near normal levels [19]. This counter regulatory mechanism most likely explains the small but significant increases in mean blood glucose concentration reflected by the increased serum fructosamine in the present studies. Fasting serum samples could not be obtained for practical reasons, and therefore it is not possible to assess standardised values of serum insulin, proinsulin, or C-peptide, which may had been used to confirm the hypothesis. An earlier study in asthmatic school children found no effect on fasting blood glucose or insulin concentration during treatment with inhaled budesonide 800 *μ*g daily from a spacer, but significantly increased insulin levels were measured during an oral glucose tolerance test [13]. This was taken to indicate that the treatment induced insulin resistance [13]. The systemic availability from a spacer device is approximately 10% compared to 20% from a Turbuhaler [22], so similar systemic activity should be expected from 800 *μ*g budesonide daily from a spacer and 400 *μ*g daily from a Turbuhaler. Thus, the results from both the studies suggest that normal or close to normal blood glucose concentrations are found during treatment with a moderate dose inhaled budesonide, but a somewhat higher insulin concentration is required to maintain normal glucose levels. Whether minute increases in mean blood glucose may have long-term clinical importance remains unknown; however, it is well known that long-term *β*-cell stress with elevated insulin production is associated with development of type II diabetes [23]. Three previous studies have been conducted in adults. Two studies reported no effects on carbohydrate turnover after treatment with inhaled budesonide 800 or 1600 *μ*g daily from a spacer in asthmatic adults [11] or after treatment with inhaled beclomethasone 2000 *μ*g daily in young healthy or elderly diabetic adults [12]. The third study that was in healthy young adults reported increased fasting insulin levels and increased blood glucose levels during oral glucose tolerance testing after inhaled beclomethasone 1000 *μ*g daily [10]. So the results from studies in adults have been conflicting. Furthermore, results obtained in adult populations should only be extrapolated to children with great caution. It was quite surprising to us that 400 *μ*g inhaled budesonide was found to cause a comparable increase in mean blood glucose to 5 mg prednisolone. The observation raises the question whether stimulatory effects may be caused by even lower doses of inhaled budesonide. Serum fructosamine reflects the average blood glucose during the last 2-3 weeks [14]. We found significant increases in serum fructosamine already after one-week glucocorticoid treatment in both trials; however, one might speculate whether larger increases would be found after 2-3 weeks treatment. 

Lipoprotein lipase is a key enzyme in the regulation of serum cholesterol and lipoproteins [24]. The activity of the enzyme is increased by glucocorticoids, which may thereby increase serum total cholesterol and HDL [24]. Increased lipoprotein lipase activity in the endothelium seems to increase the risk of arteriosclerosis, whereas increased activity in muscle and fat tissue seems to lower the risk [24]. It is currently debated whether the overall effect of increased enzyme activity is beneficial or harmful [24]. The present finding of numerically increased serum total cholesterol and HDL during prednisolone treatment may well be explained by stimulation of lipoprotein lipase. However, the observed changes in our study were small and statistically insignificant, and there are no previous data for comparison. It is highly questionable whether the small increases are insignificant due to a type 2 error. It is more likely that the changes are too small to be significant and that significant changes may occur during treatment with higher doses. Possible clinical long-term consequences should be evaluated in long-term follow up trials. We saw no changes in total cholesterol or HDL during budesonide treatment. A similar trial found small and insignificant increases in total cholesterol, but significant increases in HDL during treatment with inhaled budesonide 800 *μ*g daily but not during treatment with 400 *μ*g daily from a spacer in children [13]. In a study in healthy adults, significant increases in both total cholesterol and HDL were found during treatment with inhaled beclomethasone 1000 *μ*g daily [10], whereas others have found small but insignificant increases in total cholesterol during treatment with 2000 *μ*g daily [12]. So at present it seems fair to conclude that inhaled glucocorticoids in high doses may cause increases in total cholesterol and HDL; however, 400 *μ*g budesonide daily from a Turbuhaler does not cause such effects in school age children.

## 6. Conclusions

The present study has suggested that in children with asthma short-term treatment with prednisolone 5 mg daily or inhaled budesonide 400 *μ*g daily may increase mean blood glucose as measured by serum fructosamine. Further studies are needed to clarify possible short-term prednisolone stimulatory effects on total cholesterol and HDL. Whether our findings may have clinical implications awaits long-term follow-up trials.

## Figures and Tables

**Table 1:** Demographics presented as mean (range).

	Prednisolone trial	Budesonide trial
Number	20	17
Sex	M: 17 F: 3	M: 12 F: 5
Age/years	10.9 (7.7 to 13.8)	9.4 (7.2 to 13.4)
Tanner puberty stage	17 prepubertal 3 boys stage III	16 prepubertal 1 girl stage II
Height/cm	144.0 (112.0 to 175.0)	135.7 (114.3 to 157.6)
Weight/kg	37.2 (19.4 to 59.5)	33.1 (19.7 to 53.6)
Body mass index/kg/m^2^	17.5 (13.7 to 21.2)	17.5 (14.4 to 23.2)
BMI SDS	0.3 (−1.8 to 1.7)	0.7 (−1.2 to 3.8)

**Table 2:** Serum fructosamine, total cholesterol, and HDL during active and placebo/no treatment presented as mean (SEM).

Prednisolone trial	Prednisolone	Placebo	*P*-value	95% Cl^2^
Serum fructosamine/*μ*M^1^	252.3 [4.1]	247.3 [3.5]	0.03	0.7 to 9.3
Total cholesterol/mM^1^	4.14 [0.16]	4.00 [0.15]	0.09	−0.02 to 0.30
HDL/mM^1^	1.43 [0.08]	1.34 [0.07]	0.06	0.00 to 0.17

Budesonide trial	Budesonide	No treatment	*P*-value	95% CI^2^

Serum fructosamine/*μ*M^1^	228.1 [3.3]	223.1 [3.2]	0.02	1.0 to 9.1
Total cholesterol/mM^1^	3.99 [0.12]	3.97 [0.13]	0.76	−0.17 to 0.23
HDL/mM^1^	1.44 [0.08]	1.41 [0.06]	0.42	−0.05 to 0.10
